# Long Term Protection after Immunization with *P. berghei* Sporozoites Correlates with Sustained IFNγ Responses of Hepatic CD8+ Memory T Cells

**DOI:** 10.1371/journal.pone.0036508

**Published:** 2012-05-01

**Authors:** Krystelle Nganou-Makamdop, Geert-Jan van Gemert, Theo Arens, Cornelus C. Hermsen, Robert W. Sauerwein

**Affiliations:** Department of Medical Microbiology, Radboud University Nijmegen Medical Centre, Nijmegen, The Netherlands; Seattle Biomedical Research Institute, University of Washington, United States of America

## Abstract

Protection against *P. berghei* malaria can successfully be induced in mice by immunization with both radiation attenuated sporozoites (RAS) arresting early during liver stage development, or sporozoites combined with chloroquine chemoprophylaxis (CPS), resulting in complete intra-hepatic parasite development before killing of blood-stages by chloroquine takes place. We assessed the longevity of protective cellular immune responses by RAS and CPS *P. berghei* immunization of C57BL/6j mice. Strong effector and memory (T_EM_) CD8+ T cell responses were induced predominantly in the liver of both RAS and CPS immunized mice while CD4+ T cells with memory phenotype remained at base line levels. Compared to unprotected naïve mice, we found high sporozoite-specific IFNγ *ex vivo* responses that associated with induced levels of *in vivo* CD8+ T_EM_ cells in the liver but not spleen. Long term evaluation over a period of 9 months showed a decline of malaria-specific IFNγ responses in RAS and CPS mice that significantly correlated with loss of protection (r^2^ = 0.60, p<0.0001). The reducing IFNγ response by hepatic memory CD8+ T cells could be boosted by re-exposure to wild-type sporozoites. Our data show that sustainable protection against malaria associates with distinct intra-hepatic immune responses characterized by strong IFNγ producing CD8+ memory T cells.

## Introduction

Malaria is transmitted to the host through bites of *Plasmodium* infected mosquitoes that inject sporozoites into the skin. These sporozoites travel to the liver for further development and released as blood-stage parasites that are responsible for clinical malaria [1]. A number of whole-parasite models including sporozoites or blood-stage parasites are currently in use to study mechanisms of protective immunity [2], [3], [4]. Immunization by whole sporozoites currently makes use of three main approaches: genetically attenuated sporozoites (GAS); radiation attenuated sporozoites (RAS) or sporozoites under chemoprophylactic cover – with for instance chloroquine (CPS). RAS arrest early in the liver stage development [5], disrupting the normal cycle of the parasite while allowing the host to develop an immune response able to overcome disease upon subsequent challenge. In the CPS approach, the anti-malarial drug chloroquine (CQ) rapidly clears parasites from the bloodstream without affecting the liver stages [6] while allowing the host to mount a fully protective immune response.

Sterile protection against malaria by whole sporozoites is thought to be mediated by hepatic CD8+ T cell responses. The expansion of CD8+ T cells with memory phenotype, identified by the high expression of CD44, as well as high production of IFNγ have been shown to associate with protection by RAS [7], [8], [9], [10]. Moreover, depletion of CD8+ T cells prior to challenge have been shown to nearly entirely abrogate complete protection [11]. Regarding CPS, limited data so far suggest a protective role for both CD4+ and CD8+ T cells as well as IFNγ but not IL-6, IL-12 or TNF [6]. On top of encouraging high protection levels observed in mice studies [2], [6], [12], [13], RAS and CPS models have also been shown to induce complete protection in men [2], [14]. Better understanding of the dynamics of liver-mediated CD8+ T cell responses and evaluation on long term are essential characteristics to explore in the context of long-lived protection by a pre-erythrocytic whole parasite malaria vaccine. In the present study, we evaluate the longevity of components essential for protection by RAS or CPS immunization with *P. berghei* sporozoites.

## Results

### Protection associates with intra-hepatic effector (memory) CD8+ T cell responses

**Figure 1:**
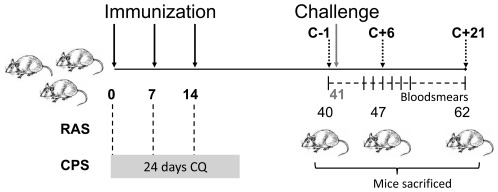
Experimental set-up high and low dose immunizations. C57BL6j mice were immunized intravenously (day 0, day 7 and day 14) by radiation attenuated sporozoites (RAS) or chloroquine prophylactic sporozoites (CPS). CPS mice received chloroquine (CQ) once a day for 24 days starting from the first day of immunization. Absence of blood-stage parasites was confirmed before challenge at day 41. Blood, liver and spleen were collected one day before challenge (C-1 – day 40), six days (C+6 – day 46) and twenty-one days (C+21 – day 62) after challenge (dotted arrows). Protection was assessed by determination of parasitemia on Giemsa stained thin blood smears between day 3 to 21 after challenge.

**Figure 2:**
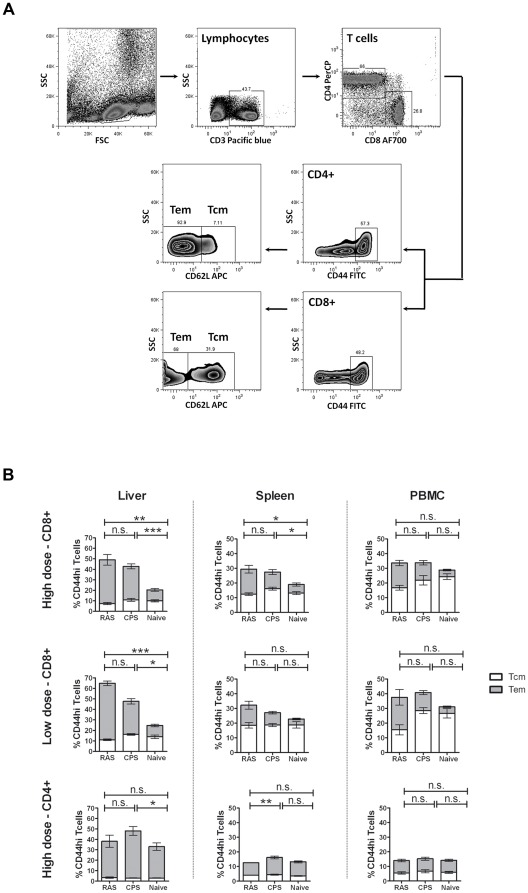
Phenotypic analyses CD44hi T-cells. (A) Gating strategy. After lymphocytes gating based on forward-scatter (FSC) and side-scatter (SSC) properties, CD4+ and CD8+ T cells were selected. Total memory T cells were gated based on high CD44 expression. T cells with effector memory (T_EM_) and central memory (T_CM_) phenotype were identified based on CD62L expression. (B) CD8+CD44hi and CD4+CD44hi T-cell pool at day 40 post-immunization with high or lose dose. Composition of the CD8+CD44hi and CD4+CD44hi T-cell pool was assessed in the liver (left panel), spleen (central panel) or PBMC (right panel) of mice immunized by high and low dose of RAS or CPS. Results are from 2 independent experiments (n_RAS_ = 10; n_CPS_ = 10; n_naïve_ = 13) and cells from individual mice assayed. Error bars represent standard error of the mean (SEM). * = p<0.05, ** = p<0.005, *** = p<0.0001.

Groups of C57BL/6j mice were immunized with either a high (50 K/20 K/20 K) or low (10 K/10 K/10 K) dose of *P. berghei* ANKA sporozoites (*Pb*spz) according to RAS or CPS protocols (Fig. 1). Induced memory responses were analyzed in PBMC, hepatic mononuclear cell (HMC) and splenocytes by flow cytometry at day 40 (26 days after the last immunization). Overall, CD4+CD44hi or CD8+CD44hi T cell responses in PBMC, HMC and splenocytes were similar for both tested regimens (Fig. 2a). Both RAS and CPS immunized groups showed a similar and clear expansion in the proportion of CD8+CD44hi T cells in liver, primarily due to an up to 4-fold increase of the CD8+ T cells with effector memory phenotype (T_EM_: CD44hiCD62L−) and without obvious alterations in the central memory (T_CM_: CD44hiCD62L+) pool (Fig. 2b). In spleen and blood, the fraction of CD8+CD44hi T cells to the total CD8+ T cell pool did not change, but there was an up to 2.5 fold proportional increase of CD8+ T_EM_ cells. Generally, the changes in CPS-induced CD8+ T_EM_ cells were smaller compared to RAS immunizations. Post-immunization changes in memory CD4+ T cell pool were minimal and only observed in HMC of immunized mice.

Both high (50 K/20 K/20 K) and low (10 K/10 K/10 K) immunization doses with either RAS or CPS regimes induced complete protection in 100% of the mice (Table 1). All naïve mice developed parasitemia as determined by Giemsa-stained smears of tail blood.

**Table 1:** RAS and CPS^a^ protection upon *P. berghei* sporozoite challenge^b^.

	No. protected/No. challenged	% protection
RAS 50/20/20 (×10^3^)	10/10	100
RAS 10/10/10 (×10^3^)	20/20	100
CPS 50/20/20 (×10^3^)	5/5	100
CPS 10/10/10 (×10^3^)	10/10	100
Naïve	0/23	0%

a CPS mice received 24-days chloroquine treatment. All immunized mice were challenged 17 days after CQ treatment.

b Cumulative data from three experiments. In two experiments, mice were challenged by i.v. injection of 10.000 WT sporozoites. In one experiment, mice were challenged by bites of 5–11 infected mosquitoes. Protection was defined as negative blood-smears at day 21 after challenge.

### Dynamics of liver CD8+ T_EM_ cells and IFNγ response in RAS and CPS immunized mice

**Figure 3:**
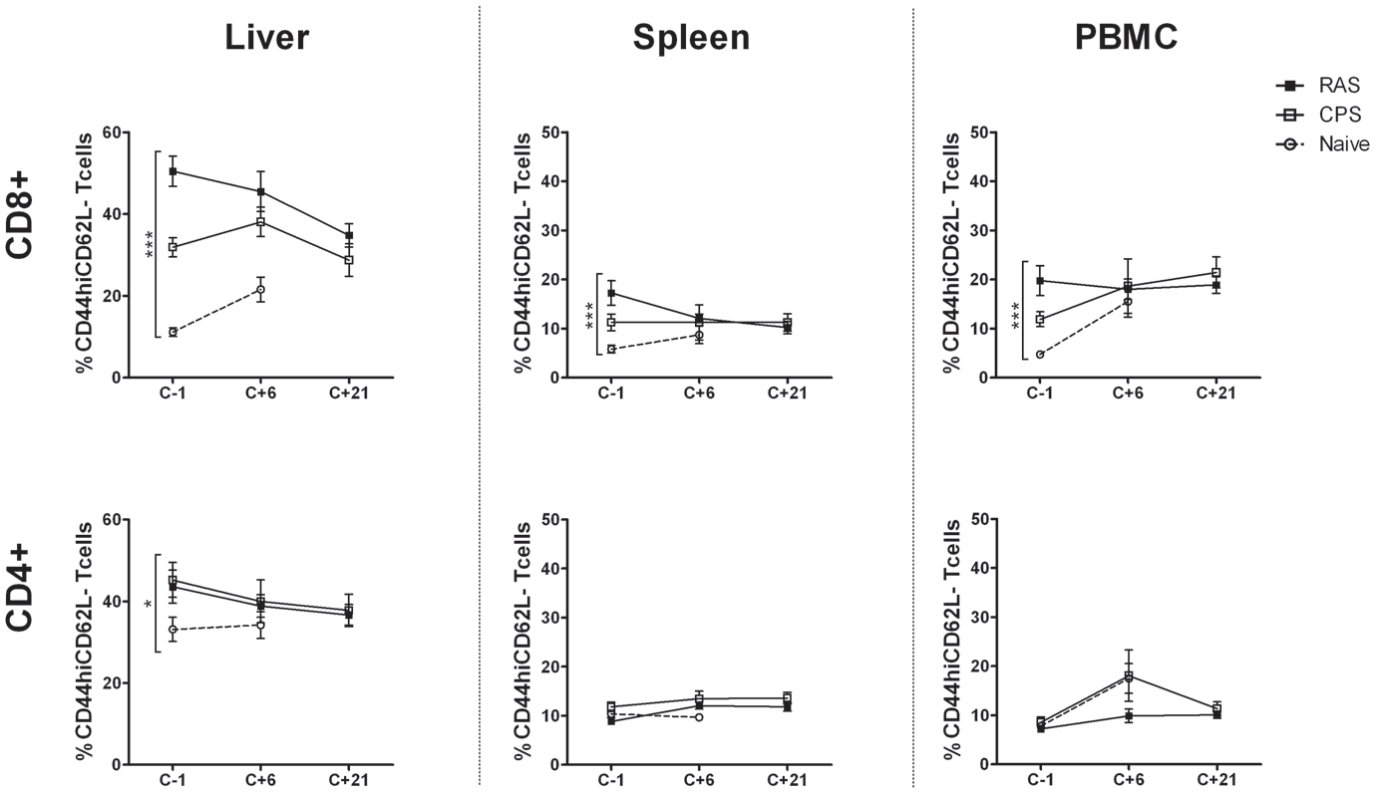
CD4+ and CD8+ T_EM_ cells in response to challenge. Changes in the effector memory CD8+ and CD4+ T-cell compartment from the liver, spleen and PBMC of mice immunized with low dose of RAS (n = 15) or CPS (n = 12) were evaluated at various time-points around challenge (C-1, C+6, C+21). Results are from 3 independent experiments (n_naïve_ = 18) and cells from individual mice assayed. Error bars represent standard error of the mean (SEM). * = p<0.05, *** = p<0.0001.

We next studied the effect of challenge infections on the dynamics of CD4+ and CD8+ T_EM_ cells. Prior to challenge (C-1) the proportion of CD8+ T_EM_ cells was higher in PBMC, HMC and splenocytes of RAS-compared to CPS immunized mice (Fig. 3). There was a gradual significant downward trend in intra-hepatic CD8+ T_EM_ cells in RAS immunized animals up to 21 days post-challenge (p = 0.007) while the post-immunization profile remained stable in CPS immunized mice. Overall, CD8+ but not CD4+ T_EM_ cells, only from liver but not spleen or peripheral blood remained significantly higher in immunized versus naïve mice. Interestingly, CD8+ T_EM_ cells from naïve mice significantly increased during fatal infection up to day 6 post-challenge (p = 0.008), most strikingly in the liver but also in spleen and peripheral blood. All data combined indicate the strongest memory T cell responses to be generated intra-hepatically.

**Figure 4:**
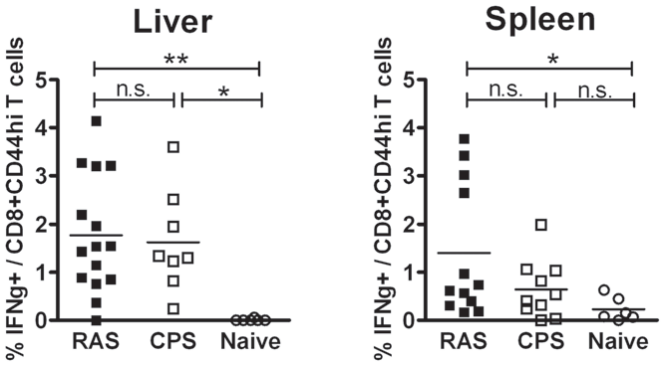
IFNγ response by liver and spleen CD8+CD44hi T cells. At day 62, liver and spleen cells collected from immune RAS or CPS mice were stimulated for 24 hours ex vivo with cryo-conserved *Pb*spz. IFNγ response was assessed by intracellular cytokine staining prior to flow-cytometry measurement (2 experiments). The percentage of IFNγ+ lymphocytes upon stimulation with PMA and ionomycin was similar between RAS (n = 15), CPS (n = 10) and naïve (n = 6) mice in the liver (6.9%, 5.2%, 5.1%) or spleen (1.33%, 1.65%, 1.1%). * = p<0.05, ** = p<0.001.

IFNγ responses of CD8+ T cells with memory phenotype (CD44hi) induced by RAS or CPS immunization was assessed 21 days following challenge at day 41 (C+21). Freshly isolated HMC and splenocytes were *ex vivo* stimulated with cryo-conserved *P. berghei* sporozoites. CD8+CD44hi T cells of RAS and CPS mice show similar IFNγ responses albeit somewhat higher in the liver than in the spleen (Fig. 4). Liver and spleen cells from naïve mice barely show any *ex vivo* IFNγ response to *Pb*spz while the positive control stimulation with PMA and ionomycin resulted in percentage of responding cells similar to immune mice (data not shown).

### Declining IFNγ response by liver CD8+ memory T cells correlates with loss of protection

**Figure 5:**
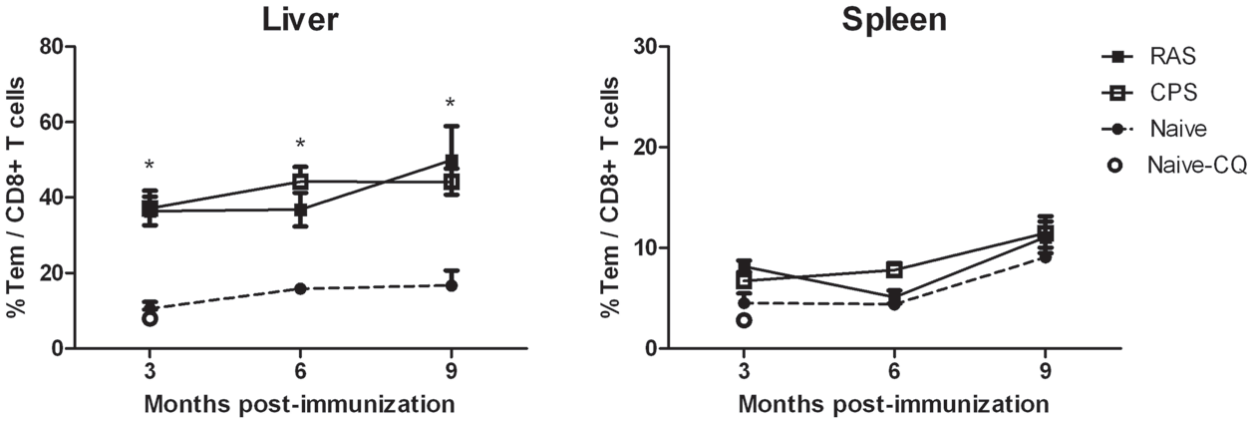
CD8+ T_EM_ levels up to 9 months post-immunization. Levels of liver and spleen CD8+ T cells with effector memory (T_EM_) were measured at 3, 6 and 9 months after RAS or CPS immunization. Error bars represent standard error of the mean (SEM). * = p<0.05.

To evaluate the sustainability of the hepatic CD8+ T cell responses measured after immunization by RAS and CPS protocols, levels of CD8+ T_EM_ were assessed 3, 6 or 9 months following the last immunization by three doses of 10 K sporozoites. Analysis of the memory pool showed compared to naïve mice, sustained and significantly high levels of CD8+ T cells with effector memory phenotype up to 9 months post- immunization (Fig. 5). In the spleen, composition of the CD8+ T cell memory pool did not differ between naïve and immunized mice.

**Figure 6:**
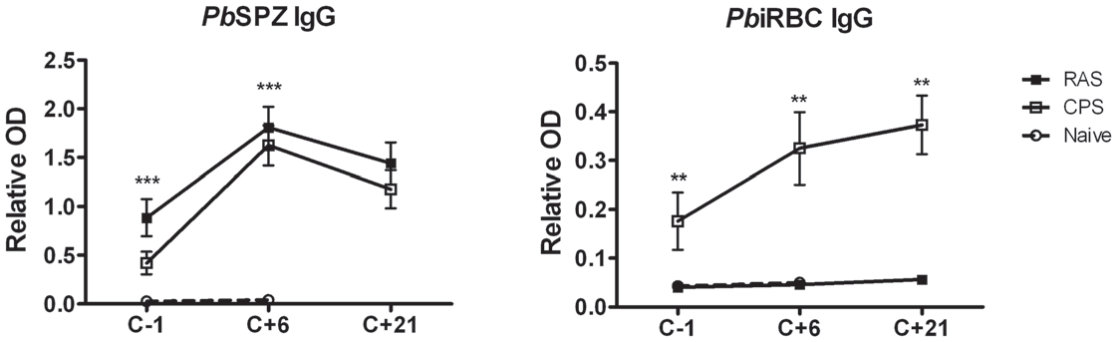
Sporozoite and blood-stage specific IgG. Plasma were collected from mice immunized by RAS or CPS before (C-1) and 6 to 21 days after (C+6; C+21) challenge. Levels of anti-sporozoite or anti-blood-stage IgG antibodies were determined by ELISA (n_RAS_ = 5; n_CPS_ = 5; n_naïve_ = 9). Error bars represent standard error of the mean (SEM). ** = p<0.005, *** = p<0.0001.

**Figure 7:**
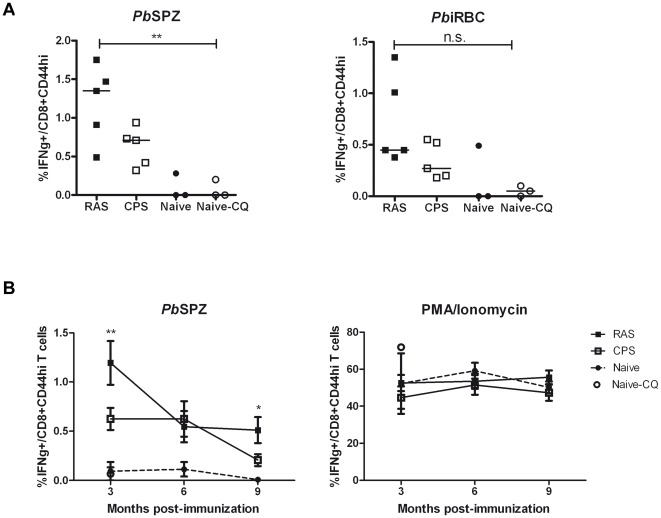
Declined specific IFNγ response by hepatic CD8+ memory T cells over time. Sporozoite specific IFNγ response by hepatic CD8+CD44hi T cells was measured by intracellular staining at 3 months post-immunization (A) – individual values and median are plotted. Longevity of the specific (sporozoites) and non-specific (PMA/Ionomycin) IFNγ response was further assessed 6 and 9 months after immunization (B) – error bars represent SEM. * = p<0.05, ** = p<0.001.

We further explored the differences in antigen exposure of RAS and CPS protocols (tested by serology) and assessed the longevity of IFNγ response by hepatic CD8+ T cells. While concentrations of anti-sporozoite specific IgG antibodies were equal in all immunized mice, only the CPS protocol as expected induced anti-blood-stage specific IgG antibodies (Fig. 6). Challenge infection resulted in a further increase of parasite specific IgG levels while RAS-induced antibodies remained negative for blood-stages. These data show that antigen exposure to RAS is limited to pre-erythrocytic stages while CPS immunization induces both pre-erythrocytic- and blood-stage specific antibodies. At 3 months post-immunization, IFNγ response by hepatic CD8+CD44hi T cells against sporozoites (*Pb*spz) or infected red blood cells (*Pb*iRBC) were significantly increased, with the highest responses in RAS immunized mice(Fig. 7A). However, a decline in *Pb*spz specific IFNγ response was observed over a 3 to 9 months post-immunization period (Fig. 7B). In contrast, non-specific polyclonal stimulation showed a constant and equally high IFNγ response at all timepoints in all groups. Bloodstage specific IFNγ response showed a similar pattern in the liver while splenocytes failed to show sporozoite or bloodstage specific IFNγ responses distinct from naïve mice (data not shown).

Long-term protection was evaluated by challenge at 3, 6 or 9 months post immunization. While RAS protocol induced 100% protection at all time points, CPS-induced protection was reduced from 100% (t = 3 and 6 months) to 50% (t = 9 months) (Table 2). Interestingly, decreased sporozoite specific IFNγ response by hepatic CD8+CD44hi T cells significantly correlated with decreased protection levels observed from 3 to 9 months (r^2^ = 0.60, p<0.0001).

**Table 2:** Long-term RAS and CPS^a^ protection following *P. berghei* sporozoite challenge^b^.

Challenge (time post-immunization)	No. protected/No. challenged (% protection)		
	RAS	CPS	Naive
3 Months	5/5 (100)	5/5 (100)	0/6 (0)
6 Months	5/5 (100)	5/5 (100)	0/3 (0)
9 Months	5/5 (100)	4/8 (50)	0/3 (0)

a CPS mice received 24-days chloroquine treatment. Three of the six naïve mice challenged at t = 3months receive the same chloroquine treatment.

b Mice were challenged by i.v. injection of 10.000 WT sporozoites. Protection was defined as negative blood-smears at day 21 after challenge.

### Re-exposure boost levels of liver CD8+ T_EM_ cells and IFNγ response

**Figure 8:**
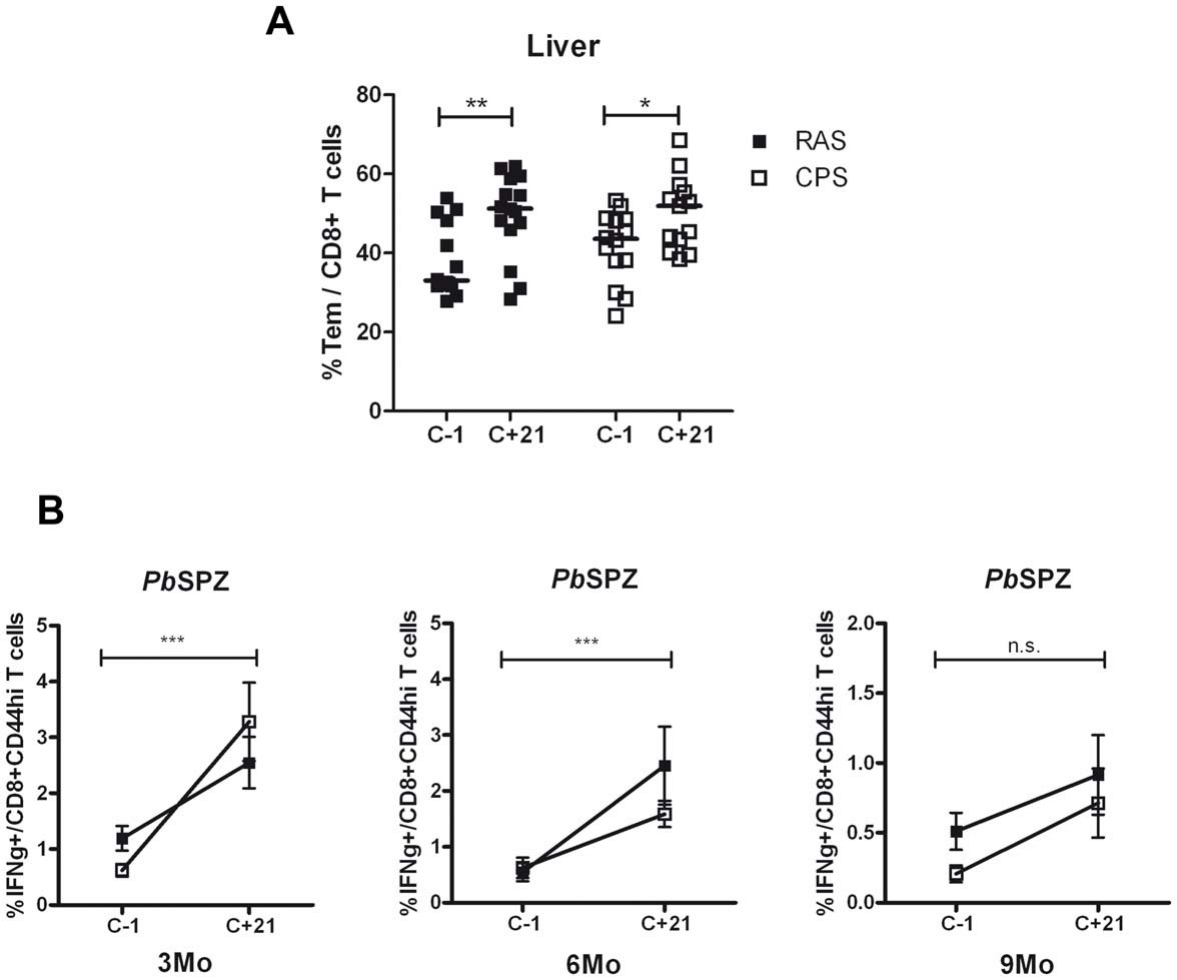
Re-exposure by sporozoite challenge increases memory CD8+ T cell response. Levels of CD8+ T_EM_ were measured before (C-1) and after (C+21) challenge at 3, 6 or 9 months after RAS and CPS immunization (A) – individual values for each time point and median are plotted. IFNγ response by hepatic CD8+CD44hi T cells was measured by intracellular staining before (C-1) and after (C+21) challenge at 3, 6 or 9 months post-immunization (B) – error bars represent SEM. * = p<0.05, ** = p<0.005, *** = p<0.0001.

We finally investigated whether decreased liver CD8+ T_EM_ cell response could be boosted by re-exposure. In protected mice challenged 3, 6 or 9 months after immunization, levels of liver CD8+ T_EM_ showed at day 21 after individual challenge infections a significant increase in proportion of CD8+ T_EM_ (Fig. 8A). Moreover, sporozoite specific IFNγ response by hepatic CD8+CD44hi T cells was boosted by each challenge infection (Fig. 8B). Although not strong, there was a significant correlation between the overall levels of CD8+ T_EM+_ cells and the sporozoite specific IFNγ response before and after challenge (p = 0.007, r2 = 0.13).

## Discussion

The present study highlights the essential role of IFNγ response by liver memory CD8+ T cell for the longevity of protection against malaria. After both *P. berghei* RAS and CPS immunization, similar T cell responses are induced with increase of predominantly the CD8+ T cell pool with memory phenotype in liver, and to a lesser extent in spleen and PBMC. The composition of the CD4+CD44hi T cell pool remains relatively unaffected. The observed changes after RAS immunization corroborate data from previous studies showing clear liver CD8+ T_EM_ cells responses with modest expansion of spleen cells and PBMC [7], [8], [9], [10]. Initially, both RAS and CPS protocols are equally efficient in the induction of complete protection, compliant with an apparent key role of liver CD8+ T_EM_ cells with IFNγ as one of the main actors. Further long term evaluation of RAS or CPS induced immune responses and protection clearly shows that up to 9 months after immunization, malaria specific IFNγ response declines despite sustained high levels of liver CD8+ T_EM_ cells. This waning IFNγ response significantly correlates with loss of protection observed in CPS mice 9 months after immunization (r^2^ = 0.60, p<0.0001). In a previous study by Jobe et al [10], *P. berghei* RAS immunization was shown to induce liver IFNγ-secreting CD8+ T cells still measurable at high levels upon re-challenge 6 months after the first challenge infection. While these findings are in line with our post-challenge observations, our pre-challenge data show that in its natural course, CD8+ T-cell mediated protective immune response declines over time yet remains detectable months after immunization prior to boost by re-exposure.

Several studies have addressed protection in murine malaria models with parallel immune responses. Schmidt et al have shown induced and sustained (up to 5 months) CD8+ memory T cell response defined by presence of CD8a^lo^CD11a^hi^ T cells in peripheral blood [15]. High levels of PBMC CD8a^lo^CD11a^hi^ T cells were however not sufficient to induce protection. Similar findings were reported by Friesen et al, showing equally high levels of PBMC CD8a^lo^CD11a^hi^ T cells in mice immunized by *P. berghei* RAS or under azythromycin chemoprophylaxis despite protection levels of 40 and 80% respectively [16]. In spite of strong indications of induced protective immune responses, abundant presence of neither CD8a^lo^CD11a^hi^ T cells (as shown by others) or CD8+ T cells with classical effector memory phenotype (CD44hiCD62L−) are sufficient to predict protection. On top of quantitative analysis of CD8+ memory T cell responses, the present study supports the predictive value of malaria specific IFNγ response for longevity of protective immunity.

Our findings of a more sustained protective immunity induced by RAS compared to CPS are somewhat surprising. As shown by Butler et al., late arresting genetically attenuated *P. yoelii* parasites induce a more robust protective immune response in mice compared to RAS [17]. Furthermore, RAS immunizations in humans are clearly less efficient than the CPS protocol [14], [18]. This may obviously be related to differences in immune mechanisms in humans and animal models using adapted species. In addition, it is difficult to make comparison between our findings and data from Butler et al. as development of genetically attenuated parasites differ between *P. berghei* and *P. yoelii* [19].

Finally, encouraging long term protection up to 42 [18] and 122 [20] weeks has been shown in humans immunized by RAS and CPS protocols respectively. Underlying protective mechanisms of RAS protection in men show on short term malaria specific IFNγ responses [21]. CPS clinical trials show that protection sustained for more than two years [14], [20] associates with malaria specific IFNγ responses that last for at least 14 months [22]. Therefore, IFNγ, presented as *‘central mediator of protective immune response against malaria’* [23], does not only point out immediate protection but could also serve as a marker for prediction of protracted protection in malaria.

## Materials and Methods

### Mice and parasites

C57BL/6j mice (6 to 8 weeks old) were purchased from Elevage-Janvier (Le Genest Saint Isle, France). Mice were housed at the Central Animal Facility in Nijmegen and received a standard diet and water *ad libitum*. All animal studies and procedures were approved by the Ethical Committee on Animal Research of the Radboud University Nijmegen (RU-DEC 2008-198, 2009-170, 2009-226).

*P. berghei* (ANKA) sporozoites (*Pb*spz) were obtained by dissection of the salivary glands of infected female *Anopheles stephensi* mosquitoes 21–29 days after blood meal on infected mice. To obtain radiation attenuated sporozoites (RAS), infected mosquitoes were irradiated at 16,000 rad (Gammacell 1000 ^137^Cs) prior to dissection.

### Immunization schedules, challenge and protection

Mice received three intravenous injections with weekly intervals of two *Pb*spz doses: 50.000-20.000-20.000 or 10.000-10.000-10.000. During CPS immunization, mice received a daily i.p. injection of 800 µg of chloroquine base starting simultaneously with the first inoculation up to two weeks after the last sporozoite inoculation. Chloroquine disphosphate (CQ, Sigma-aldrich) was diluted in PBS and administered to both immunized and naïve mice. At the end of the chloroquine treatment and approximately a day before challenge, absence of parasitemia was confirmed by examination of Giemsa-stained slides of tail blood.

Absence of blood-stage parasites prior to challenge was confirmed at the end of the rested phase in all groups. CPS or RAS immunized and naïve mice were simultaneously challenged at day 41 or 3 to 9 months after the last immunization by i.v. injection of 10,000 WT *Pb*spz. In one experiment, mice were challenged by bites of 5–11 infected mosquitoes. Presence of sporozoites in mosquitoes after feeding was confirmed by examination of the dissected salivary glands.

Parasitized red blood cells were identified by Giemsa-stained blood smears on other days from day 3–14 and finally on day 21 after challenge. Protection was defined as the absence of blood-stage parasites by day 21 post-challenge. A schematic representation of the experimental design is presented in figure 1.

### Serology

Antigen extraction from whole *P. berghei* sporozoites or infected red blood cells (iRBC) was performed as described by Bousema et al [24]. Briefly, parasites were incubated in 25 mM Tris-HCl, pH 8.0, supplemented with 150 mM NaCl, 1.0% sodium desoxycholate, and 1 mM phenylmethylsulfonylfluoride. Insoluble debris were separated by centrifugation (5 minutes, 13.000×*g* RT)and overnight coating of Sterilin ELISA plates (International Medical Products B.V.) was performed with the equivalent of 40.000 iRBC or 4.000 sporozoites per well. After blocking with 5% milk/PBS, plasma samples were incubated for three hours at room temperature (in 0.1% milk, Tween 20, PBS). Rabbit anti-mouse IgG HRP (SouthernBiotech, Birmingham, AL USA) was used for the detection of IgG antibodies to sporozoites or blood-stage antigens.

### Cell preparation

Before and after challenge, mice were euthanized by isoflurane inhalation after i.v. injection of 50 IU of heparin. Blood, spleen and liver were collected after perfusion of the liver with 10 ml of PBS. Cell suspensions of liver and spleen were made by passage of the organs through a 70-µm nylon cell strainer (BD Labware). Liver cells were resuspended in 35% Percoll (GE Healthcare) and centrifuged at 800 g for 20 min. Liver and spleen erythrocytes were lysed by 5 min incubation on ice in ACK lysing solution. After erythrocyte lysis, HMC and splenocytes were resuspended in PRMI 1640 medium. Isolation of peripheral blood mononuclear cells (PBMC) was performed using Histopaque-1077 (Sigma-Aldrich) according to the manufacturer's recommendation.

### Memory phenotyping and anti-sporozoite or bloodstage IFNγ response

Five-color staining of PBMC, HMC and splenocytes was performed using monoclonal antibodies purchased by Biolegend: Pacific blue-conjugated anti CD3 (17A2), Peridinin Chlorophyll Protein (PerCP)-conjugated anti CD4 (RM4.5), Alexa fluor 700-conjugated anti CD8a (53–6.7), fluorescein isothiocyanate (FITC)–conjugated anti-CD44, allophycocyanin (APC)– or phycoerythrin-Cy7 (PE-Cy7)-conjugated anti-CD62L (MEL-14). Briefly, 10^6^ cells were resuspended in cold assay buffer (PBS supplemented with 0.5% bovine serum albumin (Sigma-Aldrich) and incubated for 30 min at 4°C with the monoclonal antibodies. Cells were fixed with Fix & Perm medium A (Invitrogen) and resuspended in assay buffer for measurement.

HMC and splenocytes (5×10^5^ cells/well) were co-cultured in complete RPMI 1640 culture medium [25] in presence of cryo-preserved sporozoites (SPZ - 5×10^4^/ml), *P. berghei* infected red blood cells (iRBC - 5×10^6^/ml), salivary gland preparations from uninfected mosquitoes or uninfected red blood cells (uRBC - 5×10^6^/ml). Cells were stimulated at 37°C/5%CO2 for 24 hours and Brefeldin A (Sigma) was added during the last four hours (10 µg/ml final concentration). As positive control, PMA (100 ng/ml) and Ionomycin (1.25 µg/ml) (Sigma) were added simultaneously with Brefeldin A. Cells were harvested after 24-hours *in vitro* stimulation and stained with monoclonal antibodies against CD3, CD4, CD8a and CD44 as indicated above. Fixed cells were stained with APC-conjugated anti-IFNγ with Fix & Perm medium B (Invitrogen) at 4°C for 30 min.

### Flow cytometry and data analysis and statistics

Flow cytometry was performed on a 9-color Cyan ADP (Beckman Coulter) and data analysis using FlowJo software (version 9.1; Tree Star). For the analysis of cytokine production, background response to salivary glands or uRBC was subtracted from *Pbspz* and iRBC responses for each individual mouse. Overall comparisons between RAS, CPS and naïve groups was performed by Kruskas-Wallis test and subsequent individual comparisons were performed by a Dunn's multiple comparisons test using PRISM software version 5.0 (Graphpad, San Diego, CA). p<0.05 was considered statistically significant.
